# High levels of ammonia do not raise fine particle pH sufficiently to yield nitrogen oxide-dominated sulfate production

**DOI:** 10.1038/s41598-017-11704-0

**Published:** 2017-09-21

**Authors:** Hongyu Guo, Rodney J. Weber, Athanasios Nenes

**Affiliations:** 1 School of Earth and Atmospheric Sciences, Georgia Institute of Technology, Atlanta, GA 30332 USA; 2 School of Chemical and Biomolecular Engineering, Georgia Institute of Technology, Atlanta, GA 30332 USA; 3 Institute for Chemical Engineering Sciences, Foundation for Research and Technology – Hellas, Patras, GR-26504 Greece; 4 Institute for Environmental Research and Sustainable Development, National Observatory of Athens, GR-15236 P. Penteli, Greece

## Abstract

High levels of ammonia (NH_3_) have been suggested to elevate ambient particle pH levels to near neutral acidity (pH = 7), a condition that promotes rapid SO_2_ oxidation by NO_2_ to form aerosol sulfate concentration consistent with “London fog” levels. This postulation is tested using aerosol data from representative sites around the world to conduct a thorough thermodynamic analysis of aerosol pH and its sensitivity to NH_3_ levels. We find that particle pH, regardless of ammonia levels, is always acidic even for the unusually high NH_3_ levels found in Beijing (pH = 4.5) and Xi’an (pH = 5), locations where sulfate production from NO_*x*_ is proposed. Therefore, major sulfate oxidation through a NO_2_-mediated pathway is not likely in China, or any other region of the world (e.g., US, Mediterranean) where the aerosol is consistently more acidic. The limited alkalinity from the carbonate buffer in dust and seasalt can provide the only likely set of conditions where NO_2_-mediated oxidation of SO_2_ outcompetes with other well-established pathways. The mildly acidic levels associated with excessive amounts of ammonia can promote high rates of SO_2_ oxidation through transition metal chemistry, this may be an alternative important aerosol chemical contributor to the extreme pollution events.

## Introduction

pH is a fundamental particle property that affects aerosol formation, composition, toxicity and nutrient delivery^1–6^. Sulfate is a ubiquitous inorganic aerosol species that strongly regulates aerosol acidity and is produced by aqueous and gas-phase oxidation of SO_2_ along well-established pathways. Aqueous pathways dominate depending on the pH level (O_3_ under alkaline and H_2_O_2_ under acidic conditions^7^). Aqueous oxidation of HSO_3_^−^ has recently been proposed as the major mechanism of haze formation in China, but requires fine particle pH levels that are close to neutral (pH 6–7) or higher^8,9^. It is well-known that upon emission, fresh dust or seasalt particles can have a pH level that exceeds 6^10,11^, hence provide aerosol where NO_2_-mediated oxidation of sulfate is possible; however, the acidic sulfate that forms upon these particles rapidly depletes their alkaline carbonate buffer and limits any substantial NO_2_-mediated production of sulfate. Acidification is fast for submicron particles, since acidic gases (e.g., HNO_3_ and H_2_SO_4_) are rapidly scavenged by alkaline aerosols^12,13^, dust and seasalt are only minor ionic fractions compared to sulfate and nitrate^1^, and equilibrium states with gases are typically achieved within 30 minutes under ambient conditions^14–16^. The good agreements between model and observation for the semivolatile species partitioning of NH_3_-NH_4_^+^ and HNO_3_-NO_3_^−^ species, using aerosol bulk properties as model input, suggest the thermodynamic equilibrium states in many circumstances and that the ambient fine mode aerosol is consistently (and often strongly) acidic^2,17–19^. Unlike fine haze particles, fogs and cloud drops can have pH closer to neutral owing to dilution of H^+^ by the orders of magnitude more liquid water.

Wang, *et al*.^8^ and Cheng, *et al*.^9^ argue that very high levels of NH_3_ from intense agriculture (e.g., up to 50–60 ppbv in Beijing and Xi’an, China) can sufficiently elevate pH in fine mode aerosol (PM_1_ and PM_2.5_) to promote rapid sulfate formation from NO_2_ oxidation of SO_2_. We explore this by carrying out a thorough thermodynamic analysis with the ISORROPIA-II model^18^ for conditions of aerosol- and gas-phase constituents that characterize a broad range of aerosol acidities and drivers thereof. We limit our analysis to fine mode (PM_2.5_) aerosol, as the majority of the sulfate mass resides in that fraction^1,20^ (hence its pH being the most relevant for sulfate formation), and which is also the size range where thermodynamic analysis for acidity inference works best^2,17,18,21^.

## Results

To understand the major drivers of aerosol acidity, we explore pH levels for aerosol of increasing chemical complexity, and its sensitivity to NH_3_ levels found throughout the world; we focus on two well-characterized “extremes” of anthropogenic influence: the relatively clean southeastern US and the heavily polluted regions of Beijing and Xi’an, China. In our analysis, we first focus on the simplest possible composition that is atmospherically relevant: aerosol dominated by NH_4_^+^, HSO_4_^−^/SO_4_^2−^, i.e., where the effects of NO_3_^−^, Cl^−^ or nonvolatile cations (Na^+^, K^+^, Ca^2+^, Mg^2+^) is negligible. The summertime southeastern US meets this criteria, and was thoroughly studied by Weber, *et al*.^21^; the same study predicted that large amounts of NH_3_, ~ 160 µg m^−3^ (220 ppbv), is required for equilibrium with a deliquesced ammonium sulfate aerosol. Under such conditions, aerosol pH is equal to 3.2. The pH drops to about 0.1 for aerosol composed of deliquesced ammonium bisulfate, requiring a low gas-phase NH_3_ level of 0.06 µg m^−3^ (0.08 ppbv) to be in equilibrium. The transition from NH_4_HSO_4_ to (NH_4_)_2_SO_4_ aerosol increases equilibrium NH_3_ by 2700 times and aerosol acidity by roughly 3 pH units, regardless of SO_4_^2−^ level in the range of 0.1–10 µg m^−3^. Expanding the thermodynamic analysis to include the effects of other minor inorganic constituents and organic water found in the southeastern US aerosol do not change this finding; hence a 10-fold increase in NH_3_ increases aerosol pH by about one unit over a wide range of ambient NH_3_ and SO_4_^2−^ concentration (0.1–10 µg m^−3^)^21^.

**Figure 1:**
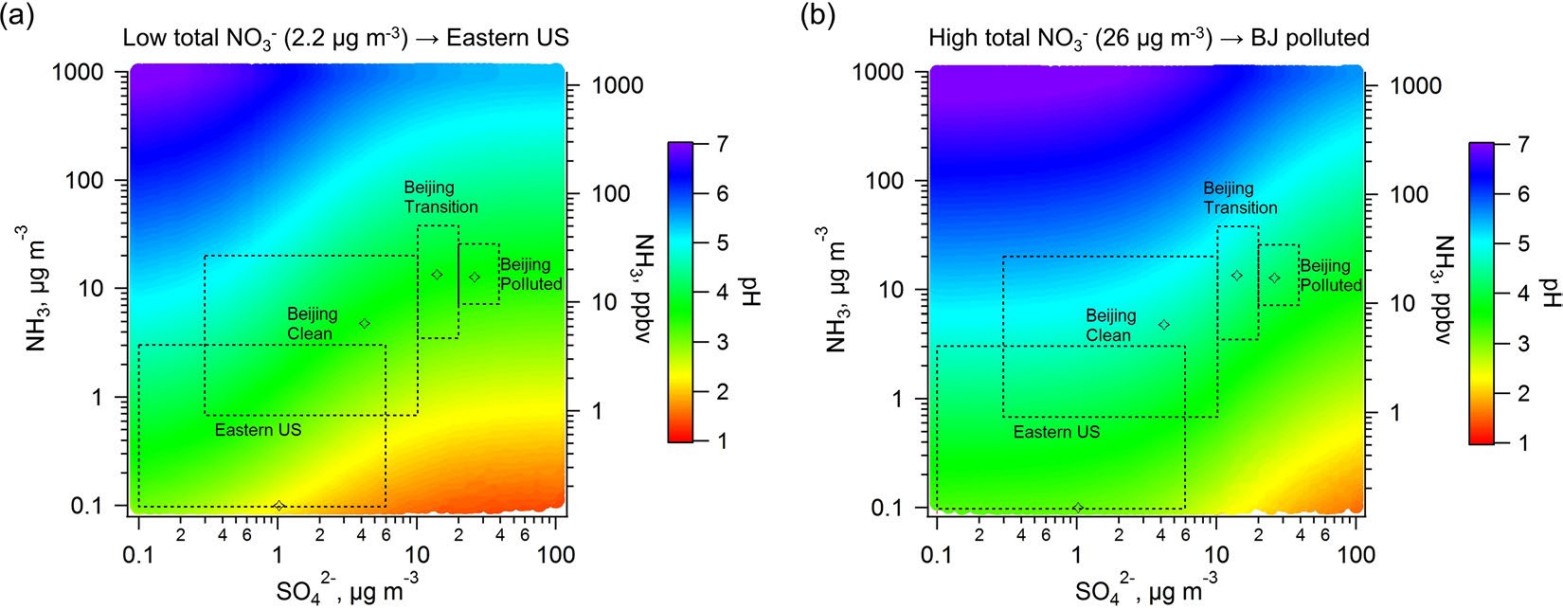
Sensitivity of PM_1_ pH to gas-phase ammonia (NH_3_) and PM_1_ sulfate (SO_4_^2−^) concentrations. The results are predictions from a thermodynamic analysis assuming equilibrium between the gas and particle phases for typical winter conditions (RH = 58%, T = 273.1 K) in (**a**) the eastern United States with low total NO_3_^−^ (HNO_3_ + NO_3_^−^) concentrations, 2.2 µg m^−3^, and (**b**) Beijing haze pollution periods with high total NO_3_^−^, 26 µg m^−3^. Boxes define observed concentration ranges for the Eastern US and Beijing and open symbols represent mean NH_3_ and SO_4_^2−^ conditions. Average total NO_3_^−^ for Eastern US, Beijing (BJ) clean, BJ transition, BJ polluted were 2.2, 6.6, 18, 26 μg m^−3^, respectively. Since total NO_3_^−^ during Beijing clean and transition periods were 6.6 µg m^−3^ and 18 µg m^−3^, respectively, graph (**a**) better represents the Beijing clean period and graph (**b**) better for the Beijing transition period.

For a more chemically-complex aerosol, where pH is controlled by the NH_4_^+^, HSO_4_^−^/SO_4_^2−^ and NO_3_^−^ system (wintertime Beijing and Xi’an meet this criteria;^8,9^), co-condensation of gas-phase NH_3_ occurs with HNO_3_ to form NH_4_NO_3_ aerosol if the ambient temperature is low enough and sufficient liquid water content is present^18^. This co-condensation also reduces the concentration of hydronium ions in the aerosol aqueous phase (i.e., increases pH) because the salts formed are less acidic than sulfate, and the additional condensed aerosol water further dilutes the aqueous phase^2,17^. The response of pH to NH_3_ in this more complex aerosol may differ from the simpler NH_4_^+^, HSO_4_^−^/SO_4_^2^ system discussed above. To study this, we carry out pH calculations for T and RH conditions representative of the eastern US and Beijing during wintertime (~0 °C and 58% RH;^2,8^) under conditions of “low” (HNO_3_ + NO_3_^−^ = 2.2 µg m^−3^, characteristic of eastern US), and “high” (HNO_3_ + NO_3_^−^ = 26 µg m^−3^; characteristic of Beijing haze) total inorganic nitrate levels. The results of the simulations are shown in Fig. 1(a,b), respectively. Regardless of total NO_3_^−^ concentration, at any SO_4_^2−^ concentration from 0.1 to 100 µg m^−3^, a 10-fold increase in NH_3_ raises pH by one unit over a wide range of NH_3_ concentrations (0.1 to 1000 µg m^−3^). In Fig. 1(a), a weak sensitivity of pH to SO_4_^2−^ is predicted for SO_4_^2−^ above 10 µg m^−3^, similar to the situation found in the southeastern US in summer^21^. For this SO_4_^2−^ range, SO_4_^2−^ mass is high enough to dominate over any effect of NO_3_^−^ on water uptake and pH, and maintains aerosol pH at 2.5 or below; for lower SO_4_^2−^ concentrations, NO_3_^−^ becomes increasingly important (for constant NH_3_) and pH increases accordingly to levels that may range between 3 and 4.5 for atmospherically-relevant levels of NH_3_. At higher levels of total nitrate (Fig. 1b), the transition from SO_4_^2–^controlled acidity (pH < 2.5) and NO_3_^–^dominant acidity (pH > 3) occurs at levels above 100 μg m^−3^ SO_4_^2−^. Therefore, for conditions of modest sulfate and high ammonia and total nitrate levels, acidity in Beijing tends to be reduced compared to the southeastern US and is largely controlled by a “nitrate-dominated” pH level.

Based on the above, the important question on what controls aerosol pH can be seen to be the relative amounts of HNO_3_ + ΝΟ_3_^−^, SO_4_, and NH_3_ + NH_4_^+^ of the system considered. The boxes indicated in Fig. 1(a,b) define characteristic areas corresponding to eastern US and Beijing aerosol (similar RH and T in winter); the pH levels inside these boxes then characterize the inherent particle acidity level of each location. The NH_3_ in the eastern US normally ranges between 0.1 and 2 µg m^−3^ with some extremes as high as 3–4 µg m^−3^ according to field measurements^22^, and the Ammonia Monitoring Network (AMoN, http://nadp.sws.uiuc.edu/amon)^23^. NH_3_ levels in Beijing were observed to be much higher, up to 38 µg m^−3^ (51 ppbv), during a heavy haze event in 2015 (Table S2 in Wang, *et al*.^8^). SO_4_^2−^ concentration in the same event reached a maximum of 38 µg m^−3^. The lowest pH is predicted for the eastern US due to the lower NH_3_ and SO_4_^2−^ compared to Beijing in Fig. 1(a,b). However, for a wide range in NH_3_ and SO_4_^2−^, particle pH for Beijing during clean, transition, and polluted periods are all around 4, and do not exceed 5. Although an extreme maximum of 300 µg m^−3^ SO_4_^2−^ was reported in another wintertime in Beijing in 2013^9^, the weak dependency of pH on SO_4_^2−^ (>10 µg m^−3^) results in a somewhat lower pH but still within the sub-100 µg m^−3^ SO_4_^2−^ ranges discussed above.

**Figure 2:**
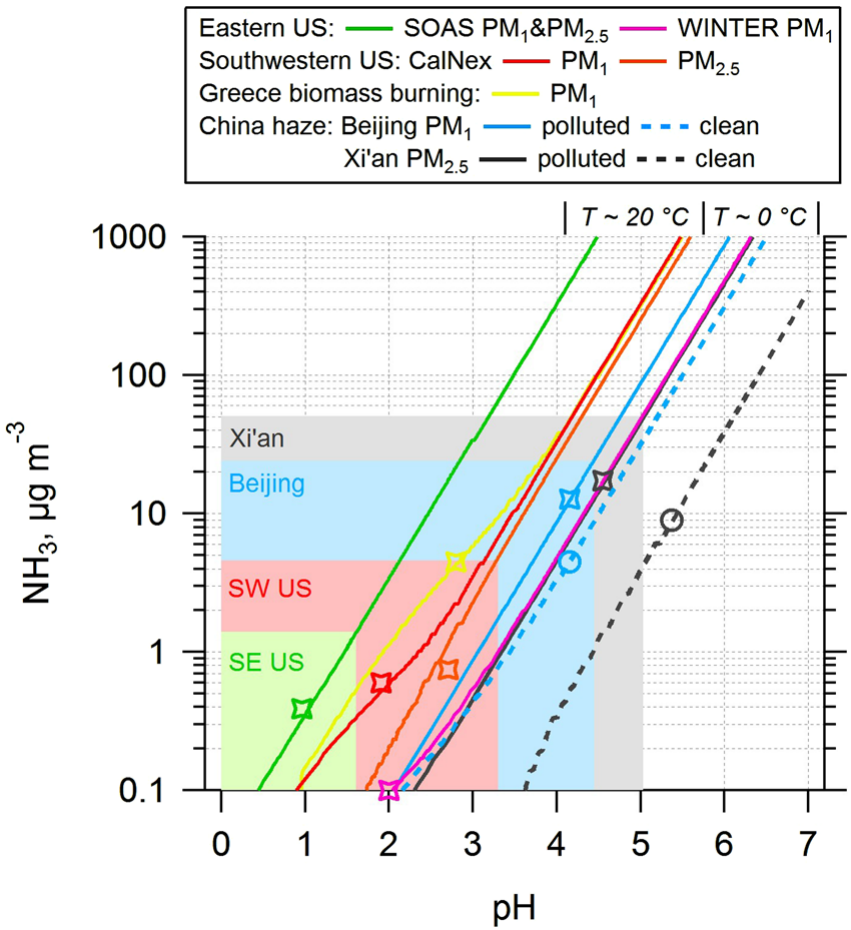
Equilibrium particle pH versus a wide range of ammonia (NH_3_) based on average aerosol and meteorological conditions (RH, T) at each site. The open symbols are the study mean pH and NH_3_, and shaded backgrounds show the upper limit of the pH range for each study (shading color matches color of study line given in the legend). Note that Xi’an polluted and WINTER PM_1_ lines overlap showing inherent consistency between the two (also true for Beijing). For the WINTER study (the only aircraft data shown), the point represents a predicted NH_3_ level 0.1 μg m^−3^ (pH = 2), whereas the reported campaign average pH (0.8 ± 1.0) is lower due to lower pH aloft (2).

The main conclusions derived from Fig. 1 do not change when the thermodynamic analysis is expanded to include a broader temperature range or the small amount of fine mode nonvolatile cations found in each region. This is shown in Fig. 2, which presents the equilibrium particle pH versus ammonia for summertime (T ~20 °C) and wintertime (T ~0 °C) conditions at different locations. Partitioning of NH_3_ and HNO_3_ towards particle-phase NH_4_^+^ and NO_3_^−^ is enhanced in lower temperatures, which as expected tends to increase particle pH. All lines become parallel for >20 µg m^−3^ NH_3_, exhibiting a sensitivity of roughly one unit pH unit increase per 10-fold increase in NH_3_. The slope of the eastern US summertime line (green) is constant throughout the entire NH_3_ range due to negligible effects of NO_3_^−^ or other nonvolatile cations on pH. The lowest range of NH_3_ and pH (0.9) is also found in the eastern US in summer. Due to the impact of high HNO_3_ and NO_3_^−^ observed in the southwest US, the lines shift to higher pH levels, despite a T, RH, and NH_3_ range similar to the eastern US. In that case the study mean PM_2.5_ pH (2.7) is nearly one unit higher than PM_1_ pH (1.9) owing to nonvolatile cations from seasalt being internally mixed with PM_2.5_, confirmed by particle mixing states measurements and thermodynamic simulations^17^. The difference between the southwestern US PM_1_ (red line) and PM_2.5_ (orange line) decreases with NH_3_, as the influence of seasalt on particle pH decreases as more and more ammonium nitrate forms. Biomass burning plumes observed in Greece reached the highest PM_1_ pH (2.8) from the effects of K^+^ and NH_3_ co-condensation with HNO_3_^24,25^ and the corresponding sensitivity line (yellow) converges with the southwestern US. Some extreme concentrations of NH_3_ (e.g. 10 µg m^−3^) in the US would increase pH to 3.5 in summer conditions. In winter conditions, although the eastern US line (purple) is very close to the Beijing lines (blue) and Xi’an polluted (black) line, the actual pH is much lower in the eastern US due to a tenfold or more lower NH_3_ concentration (on the level of 0.10 µg m^−3^); by comparison, Beijing observed on average NH_3_ 4.8 µg m^−3^ and 12.8 µg m^−3^ during clean and polluted periods respectively, and Xi’an observed even higher NH_3_ levels at 9.0 µg m^−3^ and 17.3 µg m^−3^ for clean and polluted periods. Owing to the high levels of NH_3_, the PM_1_ pH of Beijing is predicted to be 4.2 regardless of the air quality condition (clean or polluted), and the PM_2.5_ pH of Xi’an are predicted to be 4.6 and 5.4. The highest pH in Xi’an is caused by a large fraction of nonvolatile cations (Na^+^, Ca^2+^, K^+^, Mg^2+^; 31% to total aerosol ions by moles); given however that Xi’an data corresponds to PM_2.5_, and that the mixing state between the PM_1_ and PM_2.5_ can cause pH to vary up to 3 units^1^, it is likely that the aerosol pH in Xi’an exhibits a strong size-dependence that is not reflected in a simple bulk measurement and thermodynamic analysis used here. The maximum NH_3_ in Beijing and Xi’an increase pH up to 4.5 and 5.0, respectively, while the maximum NH_3_ in the southwestern US increases pH up to 3.3 in the summertime.

### Implications for sulfate formation mechanism

**Figure 3:**
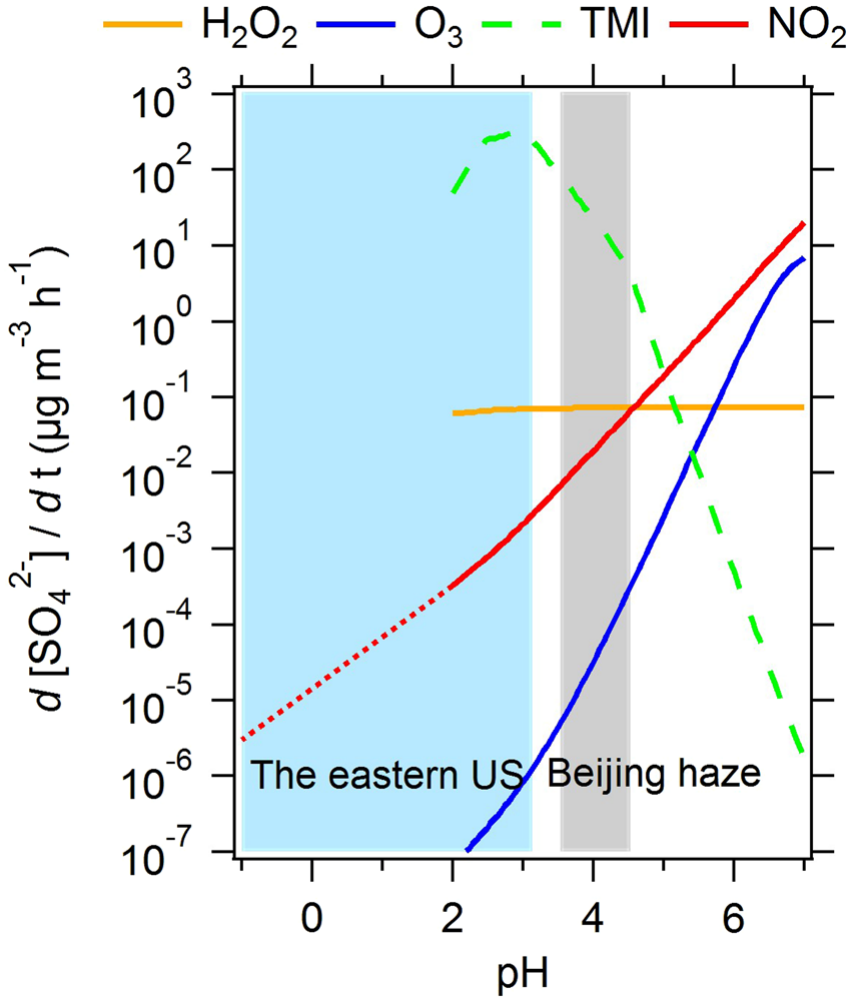
Aqueous-phase sulfate production by sulfur dioxide oxidation under characteristic conditions adapted from Cheng, *et al*.^9^ and plotted with pH ranges calculated in this study. Lines represent sulfate production rates calculated for different aqueous-phase reaction pathways with oxidants: hydrogen peroxide (H_2_O_2_), ozone (O_3_), transition metal ions (TMIs), and nitrogen dioxide (NO_2_). The gray-shaded area indicates characteristic pH ranges for aerosols during severe haze episodes in Beijing, calculated in this study. These conditions are contrasted to the lower pH of eastern US aerosol. The plot shows the NO_2_ pathway (red line) is not the main route for sulfate production.

The sensitivity of pH to NH_3_ is found to be similar between China and eastern US, despite the 10-fold or higher mass loadings of aerosols and gases of the former during intense haze pollution events. We show that for a given set of meteorological conditions (temperature and RH), roughly a 10-fold decrease in NH_3_ concentrations is required to drop pH levels by one unit, revealing an inherent consistency between vastly different aerosol systems. The pH levels between the eastern US, Beijing and Xi’an can indeed be related to the inherently different concentrations of NH_3_ found in each environment. The average pH of Beijing PM_1_ is predicted to be 4.2 (the same in clean and polluted periods), and the highest pH is about 4.5 for the maximum NH_3_ levels observed. Nonvolatile cations do not appear to considerably affect PM_2.5_ pH at Xi’an (12% mole fraction to total ions for the polluted period) compared to Beijing PM_1_, except when these cations become a large fraction of PM_2.5_ (31% mole fraction found during the clean period). Overall, Xi’an PM_2.5_ may reach a slightly higher maximum pH (5.0) than Beijing, due to even higher NH_3_ levels than Beijing. However, for all the pH ranges we find, none are in the range to provide consistent and sufficient alkalinity for the NO_2_ oxidation pathway to overwhelm sulfate formation (Fig. 3) based on the model of Cheng, *et al*.^9^. Given this, and that most of the sulfate forms where particles are most acidic (PM_1_ or PM_2.5_), it is unlikely that NO_2_-mediated oxidation of SO_2_ is a major SO_4_^2−^ formation route. Under conditions where alkalinity is sufficient to promote NO_2_ oxidation, it does not form due to the large amounts of NH_3_, but rather only from the presence of nonvolatile cations, such as those found in mineral dust and seasalt and associated carbonates that maintain pH at levels above 6. Because these species are generally limited to particles sizes larger than 1 µm diameter^1^, this route is highly unlikely to contribute to PM_1_ sulfate production, including in Beijing^20^.

The mildly acidic levels associated with excessive amounts of ammonia, however, could promote high rates of oxidation through transition metal chemistry, which overwhelms all other oxidation pathways for pH levels up to 4.5 (Fig. 3). The observed high levels of soluble transition metals that coincide with sulfate at the particle level in the PM_2.5_ range in US urban air masses^1^ and polluted air masses sampled off the coast of China^24^ supports that this may be an important pathway for explaining the high sulfate production rates, provided that the aerosol pH persists at the levels predicted here for sufficient time for the slow acid dissolution process of recalcitrant species such as iron^6^. Our analysis shows that aerosol with neutral pH is highly unlikely to be driven by excessive amounts of NH_3_; measurements of gas-phase and PM_1_/PM_2.5_ aerosol composition at rapid temporal resolution however are still required to show the frequency at which pH exceeds 4.5 during peak haze events, and whether it is possible to approach or exceed the pH 7.6 level in Beijing reported by Wang, *et al*.^8^. Our analysis also suggests this may not be likely, but measurements of size-resolved aerosol composition (including soluble transition metals) and gas-phase constituents at sufficient temporal resolution will provide the definitive observational constraints. We have shown that increasing NH_3_ does not lead to a substantially more neutral aerosol, minimizing the importance of a proposed SO_2_-NO_x_ sulfate formation route. An alternative explanation for the recent China winter haze events is changes in weather patterns that have strengthened stagnation conditions^26^.

## Methods

pH affects the equilibrated partitioning of semi-volatile compounds, such as NO_3_^−^ and NH_4_^+^, between gas- and particle-phase. Based on this sensitivity, the current most reliable method for fine particle pH is via prediction through a thermodynamic model, such as ISORROPIA-II, with gas- and particle-phase concentrations, and meteorological conditions (RH&T) as model input. ISORROPIA-II computes the equilibrium composition of an NH_4_^+^-SO_4_^2–^-NO_3_^–^-Cl^–^-Na^+^-Ca^2+^-K^+^-Mg^2+^-water inorganic aerosol (available online at: http://isorropia.eas.gatech.edu)^19,27^.

1 $${\rm{pH}}=-{\mathrm{log}}_{10}{{\gamma }}_{{{H}}^{+}}{{H}}_{{aq}}^{+}=-{\mathrm{log}}_{10}\frac{1000{{\gamma }}_{{{H}}^{+}}{{H}}_{{air}}^{+}}{{{W}}_{{i}}+{{W}}_{{o}}}\cong -{\mathrm{log}}_{10}\frac{1000{{\gamma }}_{{{H}}^{+}}{{H}}_{{air}}^{+}}{{{W}}_{{i}}}$$

pH is defined as the hydrogen ion activity in an aqueous solution^28^, where ${{\gamma }}_{{{H}}^{+}}$ is the hydronium ion activity coefficient (assumed as 1; discussed further below), ${{H}}_{\mathrm{aq}}^{+}$ (mole L^−1^) is the hydronium ion mole fraction in particle liquid water, ${{H}}_{{air}}^{+}$ (µg m^−3^) is the hydronium ion concentration per volume of air, and ${{W}}_{{i}}$ and ${{W}}_{{o}}$ (µg m^−3^) are the bulk particle water concentrations associated with inorganic and organic species, respectively. ${{W}}_{{o}}$ needs to be calculated independently by Equation (5) in Guo, *et al*.^18^, while both ${{H}}_{{air}}^{+}$ and ${{W}}_{{i}}$ are the outputs of ISORROPIA-II. Particle liquid water (${{W}}_{{i}}$+${{W}}_{{o}}$), which is essential for pH calculation, is well predicted compared to the measurement^18^. Due to a small bias between 0 and −0.2 pH often found without considering ${{W}}_{{o}}$ in the pH calculation (the logarithmic nature of pH)^2,17,18^, in this study we only calculate pH based on ${{W}}_{{i}}$, a reasonable assumption given the lower organic mass fraction reported in Beijing (on average 20–60%)^8,9^ compared to the southeastern US (on average 60%)^29^ resulting in an even smaller effect of organic particle water on pH.

ISORROPIA-II assumes ${{\gamma }}_{{{H}}^{+}}$ as unity, however, the activity coefficients of the other water-soluble ions are calculated as ionic pairs (including H^+^, e.g. H^+^-NO_3_^−^). The pH calculated from this method is proven to be similar to models that specifically calculate ${{\gamma }}_{{{H}}^{+}}$, such as E-AIM^30^, and observed and predicted gas-particle partitioning of semivolatile species are in good agreement^2,17^. We note that it is difficult to retrieve activity coefficients in concentrated aqueous solutions. The ISORROPIA-II has been tested by several ambient particle datasets with strong ionic strength, for example, the mean ionic strength 38 mole L^−1^ in the eastern US^2^. The ionic strength in Beijing haze polluted period (36 mole L^−1^) is on the same magnitude despite the much higher particle mass loadings (i.e. more particle water).

Details on how the model was run (e.g., forward mode, metastable aerosols), an extensive uncertainty analyses, and predictions of pH at various sites in the southeastern US are discussed in Guo, *et al*.^18^. The pH predictions are accurate to a high degree based on the consistency between the predicted and measured partitioning of NH_3_-NH_4_^+^ or HNO_3_-NO_3_^−^ examined in a number of studies in various locations from summer to winter conditions^2,17,18,21,24,25,30^. The thermodynamic model results are further supported by a single pair of semivolatile partitioning calculation, which appears as “S curves” and are thoroughly discussed in the section 3.6 of Guo, *et al*.^2^ and in the section 4.2 of Guo, *et al*.^17^, respectively. In applying ISORROPIA-II, we assumed no compositional dependence on particle size, treating the measured chemical constituents as bulk PM_1_ or PM_2.5_ properties, and that the aerosol (NH_4_^+^, SO_4_^2−^, NO_3_^−^) was internally mixed and composed of a single aqueous phase that contained the inorganic species, without phase separations that could affect pH (along with partitioning of semi-volatile inorganic species). In Beijing and Xi’an, the large amounts of nitrates present in the aerosol (which exhibit very low efflorescence relative humidity) and other dissolved electrolytes and organics that further depress crystallization^7^ strongly favor the presence of a single aqueous phase. pH calculated under these assumptions (bulk properties, no phase separations, dissolved components in equilibrium with the gas phase) is supported by the ability of ISORROPIA-II to reproduce independently measured gas-and particle-phase semivolatiles concentrations (e.g. NH_3_, HNO_3_, HCl). It should be noted that Wang, *et al*.^8^ heavily relied on the usage of aerosol molar ratios as a proxy of acidity, which have been shown to not represent pH well^2,18,21,30^. pH levels reported in that study were carried out with ISORROPIA-II but in stable mode and were evaluated only by predicted equilibrium NH_3_ levels by the model. Evaluation of model pH based on predicted NH_3_ (or HNO_3_) alone is insufficient because gas-phase predictions are insensitive to pH errors (Fig. S1 in the supplemental material; also shown as Fig. S3 in Guo, *et al*.^2^ for HNO_3_). Aerosol-phase concentrations, such as NH_4_^+^, NO_3_^−^, Cl^−^, are however sensitive to the assumption of phase state assumed by ISORROPIA-II and should be used for evaluation purposes (Fig. S1), which were not carried out by Wang, *et al*.^8^. When carrying out such an evaluation (Fig. S1), the metastable option reproduces aerosol NH_4_^+^, NO_3_^−^, Cl^−^ considerably better than assuming a stable aerosol, hence pH calculations from the metastable option of the model are more consistent with observed thermodynamic partitioning, hence used here. Comparing measured and predicted particle-phase fractions (e.g. ε(NH_4_^+^) = NH_4_^+^/(NH_4_^+^ + NH_3_)) provides a means for evaluation of the predicted pH. Cheng, *et al*.^9^ also carried out estimates of aerosol pH using ISORROPIA-II with the assumption of metastable aerosol, but a combination of forward and reverse-mode calculations were used; the strong dependence of pH with size^1^ and the extreme sensitivity of ammonia equilibrium vapor pressure to small errors in aerosol NH_4_^+^ when pH approaches neutral conditions^30^ also makes pH assessments that utilize reverse-mode calculations subject to considerable uncertainty.

The approach for generating the contour plots of Fig. 1 is as follows. Average RH, T, and total NO_3_^−^ (HNO_3_ + NO_3_^−^) for the eastern US or Beijing in wintertime, along with a selected sulfate concentration, are input to ISORROPIA-II. Total NH_4_^+^ (NH_3_ + NH_4_^+^) is left as the free variable. The equilibrium concentrations of various components (e.g., gas-phase NH_3_, and particle-phase NH_4_^+^, SO_4_^2−^, and NO_3_^−^) and particle pH (along with other variables) are predicted by ISORROPIA-II. Data for the contour plots are generated by varying sulfate from 0.1 to 100 µg m^−3^ while equilibrated NH_3_ covers from a wide range between 0.1 and 1000 µg m^−3^ (0.13–1333 ppbv at STP). The calculation of the sensitivity lines in Fig. 2 utilizes a simpler approach than the above due to fixed sulfate concentration at the reported campaign averages, which can be found in the supplemental material Table S1.

## Electronic supplementary material

Supplementary Information
