# Supplementary material for: High levels of ammonia do not raise fine particle pH sufficiently to yield nitrogen oxide-dominated sulfate production
Source: Sci Rep. 2017 Sep 21;7:12109. doi: 10.1038/s41598-017-11704-0 (PMC5608889; doi:10.1038/s41598-017-11704-0)
Supplement: Supplementary file 1 — Supplementary Information [file 41598_2017_11704_MOESM1_ESM.pdf]

Supporting Information for

High levels of ammonia do not raise fine particle pH sufficiently to yield nitrogen oxide-dominated sulfate production

Hongyu Guo[1], Rodney J. Weber[1*], Athanasios Nenes[1,2,3,4*]

[1] School of Earth and Atmospheric Sciences, Georgia Institute of Technology, Atlanta, GA, 30332 USA
[2] School of Chemical and Biomolecular Engineering, Georgia Institute of Technology, Atlanta, GA, 30332 USA
[3] Institute for Chemical Engineering Sciences, Foundation for Research and Technology – Hellas, Patras, GR-26504 Greece
[4] Institute for Environmental Research and Sustainable Development, National Observatory of Athens, P. Penteli, GR-15236 Greece

Correspondence to: Athanasios Nenes (athanasios.nenes@gatech.edu), Rodney J. Weber, (rweber@eas.gatech.edu)

Contents of this file

Figure S1

Table S1

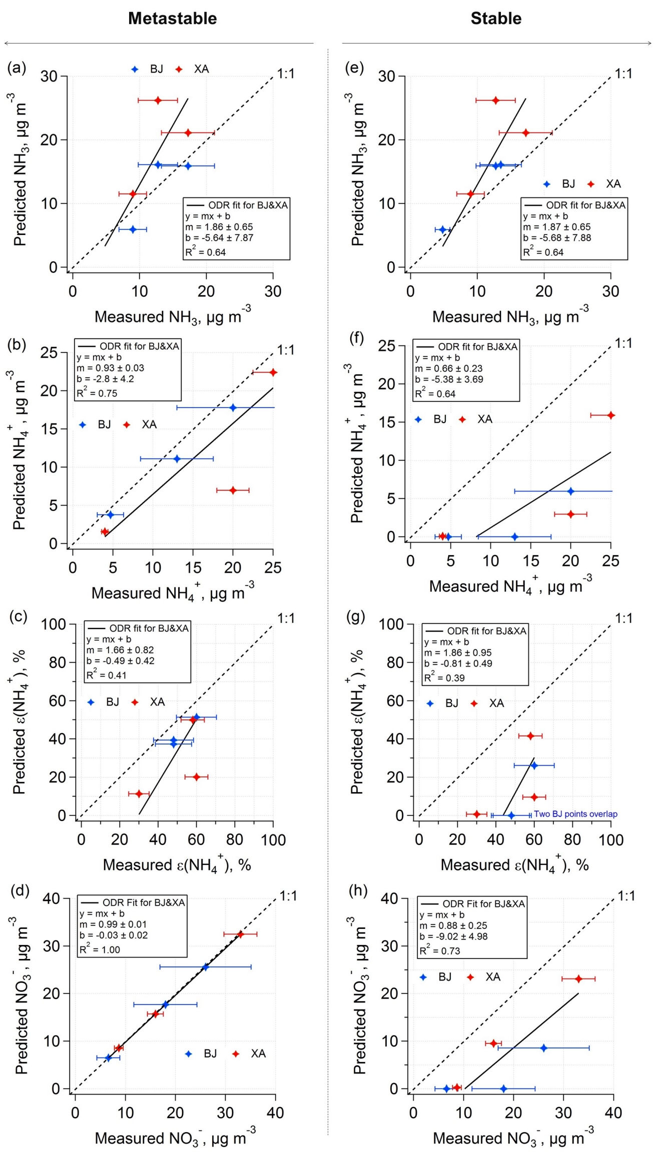

Figure S1. Comparisons of predicted and measured NH_3, NH_4^+, $\varepsilon(NH_4^+)$ (ammonium particle phase fraction, $NH_4^+/(NH_4^++NH_3)$), NO_3^- for metastable mode (left: a-d) and stable mode (right: e-h) runs in ISORROPIA-II. Data input are the average aerosol&gas concentrations and RH&T reported in Wang, *et al.* [1] (Table S1&S2) for Beijing (BJ) and Xi'an (XA) for clean, transition, and polluted periods. For HNO_3-NO_3^- partitioning, only NO_3^- is plotted for lack of HNO_3 data, which is predicted to be < 1% of NO_3^- on condition of high pH in BJ and XA. Orthogonal distance regression (ODR) fits are shown and uncertainties in the fits are one standard deviation. Measurement uncertainties are shown as error bars. Since the Wang, *et al.* [1] and related papers didn't specify the measurement uncertainties, a typical 35% AMS measurement uncertainty was used for Beijing PM_1 data [2], and a 10% measurement uncertainty assumed for Xi'an $PM_{2.5}$ data based on the MARGA methodology [3,4]. NH_3 was measured by MARGA in Beijing and Xi'an. According to Rumsey, *et al.* [3], an 23% measurement uncertainty is assumed for NH_3. The uncertainties in $\varepsilon(NH_4)^+$ are calculated based on error propagation of the NH_3 and NH_4^+ measurements.

Table S1. The study average aerosol composition and meteorological conditions for Figure 2 ISORROPIA-II input. The reported concentrations and RH, T are based on field measurements unless noted specifically. Total NH_4^+ (NH_3+NH_4^+) is left as the free variable for ISORROPIA-II calculations. The high pH predicted at Beijing and Xi'an indicate > 99% total NO_3^- (HNO_3+NO_3^-) is NO_3^-, so no report of HNO_3 data causes negligible bias.

Region/Location	SE US	NE US	SW US		Greece	Beijing, China		Xi'an, China	
Campaign	SOAS	WINTER	CalNex		(Biomass Burning)[d]	(Clean)	(Polluted)	(Clean)	(Polluted)
Sampling type	Ground	Aircraft	Ground	Ground	Ground	Ground		Ground	
PM cut size	PM_1&$PM_{2.5}$[a]	PM_1	PM_1	$PM_{2.5}$[c]	PM_1	PM_1		$PM_{2.5}$	
Year	2013	2015	2010		2012&2014	2013		2013	
Season	Summer	Winter	(Early) Summer		Summer&Winter	Winter		Winter	
Na^+, μg m^{-3}	0.03	0	0	0.77	0.08	0	0	3.6	4.2
SO_4^{2-}, μg m^{-3}	1.73	1.02	2.86	1.88	1.66	4.2	14	5.9	38
Total NH_4^+, μg m^{-3}	0.78	0.50	3.44	2.54	1.02	9.5	33.5	13	44.3
Total NO_3^-, μg m^{-3}	0.45	2.21	10.22	8.19	3.36	6.6	18	8.7	33
Cl^-, μg m^{-3}	0.02	0	0	0.64	0.20	0.8	1.6	4.0	14
Ca^{2+}, μg m^{-3}	0	0	0	0	0	0	0	1.6	2.3
K^+, μg m^{-3}	0	0	0	0	0.36	0	0	1.3	4.6
Mg^{2+}, μg m^{-3}	0	0	0	0	0	0	0	0.2	0.3
RH, %	74	58	79	87	68	40[e]	56	46	68
T, °C	25	0	18	18	20	0.4	0.9	5.7	4.1
NH_4^+, μg m^{-3}	0.46	0.50	2.06	1.79	1.02	4.7	20	4.0	25
NH_3, μg m^{-3}	0.39	0.10[b]	1.37	0.75	\	4.8	13.5	9.0	17.3
NO_3^-, μg m^{-3}	0.08	0.80	3.58	3.74	1.79	6.6	18	8.7	33
HNO_3, μg m^{-3}	0.36	1.41	6.65	4.45	0.91	\	\	\	\
Reported pH	0.9 ± 0.6	0.8 ± 1.0	1.9 ± 0.5	2.7 ± 0.3	2.8 ± 0.6	\	\	\	\
Reference	Guo, *et al.* [5]	Guo, *et al.* [6]	Guo, *et al.* [7]		Bougiatioti, *et al.* [8, 9]	Wang, *et al.* [1]		Wang, *et al.* [1]	

ISORROPIA-II input

[a] $PM_{2.5}$ was sampled in the 1st half and PM_1 sampled in the 2nd half of the study; various parameters were similar in both cases, crustal components were higher in $PM_{2.5}$, but generally low so differences had minor effects, e.g., $PM_{2.5}$ Na^+ was 0.06 ± 0.09 μg m^{-3} and PM_1 Na^+ was 0.01 ± 0.01 μg m^{-3}; [b] Prediction based on iteration; [c] Only the last week of CalNex; [d] Averaged from the identified biomass burning plumes from Crete and Athens studies due to the similar pH; [e] The reported 21% RH was too low for a completely aqueous aerosol, therefore, not suitable for discussion on pH and pH affected aqueous reactions in this study. An assumed 40% RH is applied, as the efflorescence RH of ammonium sulfate is right below 40% [10].

Reference:

1 Wang, G. *et al.* Persistent sulfate formation from London Fog to Chinese haze. *Proc Natl Acad Sci USA* **113**, 13630-13635, doi:10.1073/pnas.1616540113 (2016).

2 Bahreini, R. *et al.* Organic aerosol formation in urban and industrial plumes near Houston and Dallas, Texas. *Journal of Geophysical Research* **114**, D00F16, doi:10.1029/2008jd011493 (2009).

3 Rumsey, I. C. *et al.* An assessment of the performance of the Monitor for AeRosols and GAses in ambient air (MARGA): a semi-continuous method for soluble compounds. *Atm. Chem. Phys.* **14**, 5639-5658, doi:10.5194/acp-14-5639-2014 (2014).

4 Makkonen, U. *et al.* Semi-continuous gas and inorganic aerosol measurements at a Finnish urban site: comparisons with filters, nitrogen in aerosol and gas phases, and aerosol acidity. *Atm. Chem. Phys.* **12**, 5617-5631, doi:10.5194/acp-12-5617-2012 (2012).

5 Guo, H. *et al.* Fine-particle water and pH in the southeastern United States. *Atm. Chem. Phys.* **15**, 5211-5228, doi:10.5194/acp-15-5211-2015 (2015).

6 Guo, H. *et al.* Fine particle pH and the partitioning of nitric acid during winter in the northeastern United States. *Journal of Geophysical Research: Atmospheres* **121**, 10355-10376, doi:10.1002/2016jd025311 (2016).

7 Guo, H. *et al.* Fine particle pH and gas–particle phase partitioning of inorganic species in Pasadena, California, during the 2010 CalNex campaign. *Atm. Chem. Phys.* **17**, 5703-5719, doi:10.5194/acp-17-5703-2017 (2017).

8 Bougiatioti, A. *et al.* The unappreciated effects of biomass burning on fine mode aerosol acidity, water and nitrogen partitioning. *In review* (2017).

9 Bougiatioti, A. *et al.* Particle water and pH in the Eastern Mediterranean: Sources variability and implications for nutrients availability. *Atm. Chem. Phys.* **16**, 4579-4591, doi:10.5194/acp-16-4579-2016 (2016).

10 Tang, I. N. & Munkelwitz, H. R. Water Activities, Densities, and Refractive-Indexes of Aqueous Sulfates and Sodium-Nitrate Droplets of Atmospheric Importance. *J Geophys Res-Atmos* **99**, 18801-18808, doi:10.1029/94jd01345 (1994).
